# Vaccination in the Army

**Published:** 1898-10

**Authors:** Montague R. Leverson

**Affiliations:** Fort Hamilton, N. Y.


### VACCINATION IN THE ARMY.

FORT HAMILTON, N. Y., September 8th, 1898.

EDITOR THE HOMŒOPATHIC PHYSICIAN.

DEAR SIR:—I desire, through your pages, to entreat as many as possible of your readers to write to the President or to the Secretary of War, urging upon their attention the fact that one, if not the chief of the causes of the breakdown of our army is the vitality of the boys having been lowered by the pouring into their blood *the putrefying matter of a sore;* for every one of them was "vaccinated," as a preparation (?) for the hardships of a campaign in a malarious climate.

If forty or fifty respectable phyicians in good standing will write to the President and Secretary of War hereon they *must* take notice of it and *investigate*.

Besides, as it will tend to throw off of their shoulders some of the awful responsibility they have incurred, they will doubtless be glad to do so, if once satisfied that there *may* be something in it.

Respectfully,

MONTAGUE R. LEVERSON.

---

